# The Hypervariable Region of K-Ras4B Governs Molecular Recognition and Function

**DOI:** 10.3390/ijms20225718

**Published:** 2019-11-14

**Authors:** Hazem Abdelkarim, Avik Banerjee, Patrick Grudzien, Nicholas Leschinsky, Mahmoud Abushaer, Vadim Gaponenko

**Affiliations:** 1 Department of Biochemistry and Molecular Genetics, College of Medicine, University of Illinois at Chicago (UIC), Chicago, IL 60607, USA; habdel3@uic.edu (H.A.); pgrudz2@uic.edu (P.G.); nlesch3@uic.edu (N.L.); mabush4@uic.edu (M.A.); 2 Department of Chemistry, University of Illinois at Chicago (UIC), Chicago, IL 60612, USA; abaner7@uic.edu

**Keywords:** K-Ras4B, GTPases, hypervariable region, posttranslational modifications, autoinhibition, membrane binding motifs, protein–protein interactions

## Abstract

The flexible C-terminal hypervariable region distinguishes K-Ras4B, an important proto-oncogenic GTPase, from other Ras GTPases. This unique lysine-rich portion of the protein harbors sites for post-translational modification, including cysteine prenylation, carboxymethylation, phosphorylation, and likely many others. The functions of the hypervariable region are diverse, ranging from anchoring K-Ras4B at the plasma membrane to sampling potentially auto-inhibitory binding sites in its GTPase domain and participating in isoform-specific protein–protein interactions and signaling. Despite much research, there are still many questions about the hypervariable region of K-Ras4B. For example, mechanistic details of its interaction with plasma membrane lipids and with the GTPase domain require further clarification. The roles of the hypervariable region in K-Ras4B-specific protein–protein interactions and signaling are incompletely defined. It is also unclear why post-translational modifications frequently found in protein polylysine domains, such as acetylation, glycation, and carbamoylation, have not been observed in K-Ras4B. Expanding knowledge of the hypervariable region will likely drive the development of novel highly-efficient and selective inhibitors of K-Ras4B that are urgently needed by cancer patients.

## 1. Introduction

To perform their function, proteins often engage in interactions with their partners. These binding partners can be proteins, lipids, nucleic acids, carbohydrates, or other types of molecules. Often, binding events come with a significant entropic penalty, especially if the proteins use their flexible regions to establish intermolecular contacts. Although, this high entropic penalty can be compensated by an enthalpic contribution to allow high-affinity binding. Alternatively, the flexible regions could fine-tune thermodynamics of binding by generating entropy [1].

One example of a highly flexible region that mediates binding is the C-terminal hypervariable extension of K-Ras4B, an important GTPase that is frequently mutated in lung, colorectal, and pancreatic cancer [2,3,4,5]. This region distinguishes K-Ras4B from other Ras proteins and is comprised mostly of cationic amino acids with the final C-terminal cysteine bearing prenyl (either farnesyl or geranylgeranyl) and methyl groups. The hypervariable region (HVR) of K-Ras4B has initially been identified as a plasma membrane targeting element, in which the poly-basic stretch is attracted to the anionic phospholipids and the prenyl group inserts into the bilayer [6]. The absence of palmitoylation in the HVR is a unique characteristic of K-Ras4B, allowing its preferential localization in disordered lipid microdomains, while palmitoylated Ras GTPases primarily associate with lipid rafts [7]. This peculiar membrane binding of K-Ras4B allows it to access specific effectors and dictates unique functional outcomes. Interference with association of K-Ras4B with the plasma membrane either via inhibition of prenylation or via competition for membrane binding sites with small molecules abrogates signaling and has been extensively used to develop anti-cancer therapeutics [8]. These efforts are still ongoing, since there are no direct inhibitors of K-Ras4B in clinical use [9].

In addition to membrane targeting, emerging evidence supports involvement of HVR in intramolecular interactions with the G-domain of K-Ras4B [10] and in intermolecular association with other proteins, including farnesyltransferase [11], tubulin [12], phosphodiesterase δ (PDE-δ) [13], calmodulin [14], and likely many others. Because it is unique among Ras GTPases, the HVR of K-Ras4B, through specific intra- and intermolecular interactions, imparts distinct functional characteristics to this protein, affecting its regulation and signaling. The presence of PKC and PKA phosphorylation sites in the HVR [15] allows modulation of interaction with the plasma membrane [16,17] and binding to calmodulin [18]. Whether the HVR is also regulated by dephosphorylation is unknown and possible phosphatases for this dephosphorylation have not been identified. While most efforts have focused on characterization of HVR binding to the plasma membrane, its participation in protein–protein interactions and modulation of these interactions by post-translational modifications are emerging areas of research. We anticipate significant expansion of these areas in the near future.

In this review, we discuss how the HVR of K-Ras4B is post-translationally modified and how it establishes interactions with plasma membrane lipids, with the G-domain, and with other proteins. Given K-Ras4B’s ability to activate unique signaling pathways, we predict future identification of novel post-translational modifications in the HVR as well as discovery of K-Ras4B’s binding partners, with which the HVR selectively interacts. We expect that this knowledge will significantly advance the understanding of K-Ras4B signaling and provide insight into its therapeutic targeting in cancer.

## 2. The HVR Interacts with the G-domain

**Figure 1:**
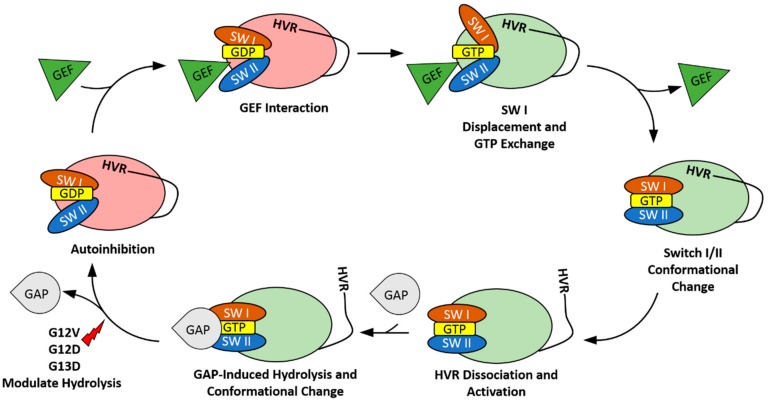
K-Ras4B auto-inhibition by the hypervariable region (HVR). Sequestration of the HVR by K-Ras4B is regulated by the bound nucleotide. In the GDP-bound state, the HVR can form extensive interactions with the G-domain, leading to an autoinhibited state. However, the exact mechanism of autoinhibition release has yet to be determined. The current working model hypothesizes that HVR release occurs following nucleotide exchange and switch I/II conformational change. It is worth noting that oncogenic mutations (red lightning bolt) in K-Ras4B such as G12V and G12D modulate the GTPase activating proteins (GAP) induced/intrinsic return to the autoinhibited state. SW I refers to the switch I region, SW II refers to the switch II region, GEF refers to guanine nucleotide exchange factors, GDP-bound G-domains are represented in pink, and GTP-bound G-domains are represented in light green.

The classical mechanism for small GTPases, such as Ras, dictates that biological activity is controlled by the presence of bound GDP or GTP. In the GDP-bound form, K-Ras4B exists in a conformation where its Switch I and Switch II regions sterically hinder effectors from accessing their binding sites. Upon the introduction of GTP through the actions of guanine nucleotide exchange factors (GEFs), the G-domain undergoes conformational changes in Switch I and Switch II regions, facilitating effector binding [19]. GEFs, such as Son-of-Sevenless (SOS), prefer to bind to the switch II region and stabilize the open, nucleotide-free state by displacing switch I [20] (Figure 1). This prevents interactions between the phosphate groups of the nucleotide and the magnesium ion. The high concentration of cytosolic GTP then allows GTP loading and activation of Ras following GEF release. Effectors then initiate signaling events until the combined effects of GTPase activating proteins (GAPs) and Ras’s intrinsic GTP hydrolysis return the enzyme to its GDP-bound inactive state (Figure 1). However, recent studies have shown that K-Ras4B can modulate this activity using inter-domain interactions. The C-terminal HVR of K-Ras4B has been a prime candidate implicated in many of these interactions. The conformational changes resulting from these contacts stabilize the protein in a variety of orientations that facilitate or restrict activity beyond the nucleotide loading [10,21,22]. Furthermore, these interactions can impact the intrinsic GDP/GTP characteristics of the enzyme. The importance of the HVR in K-Ras4B behavior is also highlighted through the particularly disruptive effects seen when mutations manifest in this region.

K-Ras4B predominantly exists in the cell in a GDP-bound state. In this state, the HVR can establish multiple contacts with the G-domain [22]. These interactions form a surface parallel to the membrane, which occludes the effector binding region immediately downstream of Switch I and results in auto-inhibition (Figure 1) [23]. This hinders interactions with effectors, such as Raf and PI3K. The exact mechanism of HVR autoinhibition release is not yet known, but it probably includes cooperative effects of GEFs, GAPs, effectors, and the membrane. GDP-induced HVR contacts are likely transient, which suggests that there are many forms of interaction between the two domains [10,22]. This stems from two distinct structural areas of the HVR: A less flexible N-terminal region (175–180 aa) extending from α5 and a highly flexible C-terminal region (181–184 aa) [24,25]. The formation of coiled or stranded structures in the rigid portion dictates the G-domain location the HVR will interact with, while the flexible tail modulates the Switch I/β2 region. These secondary structures are sequence-dependent and could explain the differences in Raf binding between splice variants [10]. In addition to blocking effector binding, HVR can slow the actions of GEFs. This occurs similarly to the regulation of Raf, as the GEF binding site is also affected by the HVR binding motif. However, this effect is significantly less pronounced than that seen in Raf and only slows GEF activity by approximately 35% [10]. This additional regulation is not seen in HVR-truncated K-Ras4B but can be rescued through the introduction of a soluble HVR peptide analog [10].

Upon exchange of GDP for GTP, the interactions between the G-domain and the HVR are weakened. This causes release of the HVR where it then orients itself in a semi-perpendicular direction to the membrane [23]. The resulting conformational changes enable effector access to the binding sites located on the β2 strand. However, it should be noted that, in molecular dynamics simulations, the HVR continues to interact with the G-domain in the GTP-bound state, albeit to a lesser extent [10]. Mutations that result in an alteration of HVR positioning have been implicated in a preference for the GTP bound state over the GDP bound state. Mutations in the rigid region of the HVR are especially disruptive to the autoregulation of K-Ras4B and induce significant differences in its activity, as compared to mutations in the more flexible region. In the case of the D173P point mutation, secondary HVR structure was completely disrupted and interrupted the HVR/G-domain interactions. However, the K180A and K182A mutations primarily affected the Switch I region of the enzyme [10]. Furthermore, common oncogenic mutations occurring within the P-loop, such as G12V and G12D, have also been shown to be capable of long-range disruption of the HVR [26]. These observations have been correlated with a reduction in the formation of K-Ras4B dimers and has led to the proposition that the HVR plays a role in the formation of dimers or higher order oligomers [27,28]. Stabilization of the HVR-autoinhibited state in oncogenic K-Ras4B presents an appealing pathway for structure-based drug design. Thus, the HVR can be critical for both controlling the activity of K-Ras4B as well as modulating its oncogenic properties.

## 3. The HVR Participates in Protein–Protein Interactions

HVR of K-Ras4B mediates protein–protein interactions. Perhaps the earliest evidence of this comes from the X-ray structure of the farnesyltransferase-HVR peptide complex [11]. This structure revealed participation of C-terminal lysines in K-Ras4B in binding farnesyltransferase and explained the high efficiency of farnesylation of this Ras isoform in comparison with other Ras GTPases.

The role of HVR in protein–protein interactions is further exemplified by the X-ray structure of fully processed K-Ras4B complexed with PDE-δ [13]. In this structure, the HVR exhibits its ability to form an α-helix, while its KSKTK amino acid sequence upstream of the polylysine region makes the PDE-δ recognition site, inserting the farnesyl group into the PDE-δ hydrophobic pocket. This arrangement facilitates extraction of K-Ras4B from the plasma membrane by PDE-δ [29]. Similarly, the calcium sensor protein calmodulin selectively extracts K-Ras4B from the plasma membrane [30,31,32] but does not interact with other Ras proteins [33]. This interaction is primarily mediated by the HVR [14,34], while under some conditions, the G-domain also supports binding to calmodulin [18,35,36]. The K-Ras4B-calmodulin complex plays diverse functional roles. It redistributes K-Ras4B from the plasma membrane to the endosomal compartment and Golgi [37], abates Raf signaling [38], suppresses the non-canonical branch of Wnt signaling [39], and potentially stabilizes the interaction of K-Ras4B with PI3K [40]. The polybasic patch within the HVR is responsible for K-Ras4B binding to the chaperone protein SmgGDS [41]. This chaperone escorts newly synthesized K-Ras4B to cytosolic prenyltransferases and signaling cascades and small molecules affecting SmgGDS could regulate prenylation and membrane binding of K-Ras4B [42]. A similar role of HVR in K-Ras4B trafficking to the plasma membrane is mediated through its interactions with tubulin, where HVR’s lysines and the carboxymethyl group are major contributors to binding [12]. Importantly, the polybasic region of the HVR facilitates selective binding of K-Ras4B to B-Raf through the anionic N-terminus of this kinase [43]. Thus, HVR-mediated protein–protein complexes play a critical role in Ras isoform-specific trafficking and signaling.

There are several more examples of selective binding of K-Ras4B to other proteins. These include the scaffolding protein Sur8 [44,45], nucleolar and membrane associated proteins nucleophosmin and nucleolin [46], a lectin family member galectin 3 [47], vacuolar v-ATPase a2 and translation initiation factor eIF2Bδ [48], and phosphatidylinositol-4-phosphate 5-kinase type 1α [49]. However, it is currently unknown whether the HVR participates in interactions with these proteins. Although the HVR is often the primary suspect as a key mediator of isoform-specific protein–protein association, there might be other players. For instance, scaffolding proteins of the RASSF family can be Ras isoform selective with RASSF1A and RASSF2 preferentially interacting with K-Ras4B [50,51]; but K-Ras4B-RASSF interactions primarily engage K-Ras4B’s G-domain [52]. Although highly homologous, the G-domains are not identical among Ras GTPases and can potentially be involved in isoform-specific protein–protein complexes and signaling. Another important aspect of studying Ras isoform-specific binding relates to verification of these interactions using endogenously expressed proteins. For example, the IQ motif GTPase activating protein 1 (IQGAP1) was found to specifically bind K-Ras4B when either protein was ectopically expressed [53]. However, more recent studies found that IQGAP proteins are functional interactors of Ras under the conditions of their endogenous expression [53]. Thus, more research is needed to more clearly define the role of HVR in selective binding of K-Ras4B to its partners. It is critical to keep in mind that Ras isoform—specific interactions are not necessarily mediated by the HVR and may not be functionally relevant.

## 4. The HVR Binds to the Plasma Membrane

Ras family proteins rely heavily on the phospholipid binding to propagate signals. However, unlike its counterparts N-Ras, H-Ras, and K-Ras4A, which have several post-translational modifications to stabilize binding, K-Ras4B utilizes the poly-basic domain of the HVR and the farnesyl group to bind lipids. K-Ras4B tethers to the membrane using a mixture of van der Waals interactions between its farnesylated residue and the phospholipid tail. This tethering is further strengthened through binding between the HVR, specifically the poly-basic domain, and the anionic lipids [24,25]. The details of these interactions, especially the HVR, are relatively unknown but there have been many studies that have begun to explore the intricacies of the HVR and how it impacts the phospholipid binding.

The poly-basic domain, which consists of eight lysine residues, has been the focal point of studies that examine the interactions of K-Ras4B with phospholipids. The importance of the HVR can be seen when testing phospholipid binding with a full-length vs. truncated HVR. The truncation severely impacts the strength of binding to certain phospholipids and decreases G-domain interactions. Thus, the HVR not only allows for more binding sites but opens the possibility of long-range interactions, which disappear when the HVR is truncated [54]. The phospholipid population within the membrane is imperative for plasma membrane anchoring. Poly-basic domain containing proteins that are targeted to the plasma membrane can only successfully detach after depletion of PIP2 and PIP3, suggesting that both phosphatidylinositols are required for the efficient membrane anchoring [55]. K-Ras4B G12V/G12D oncogenic mutants also bind to phosphatidic acid (PA), suggesting that the change in phospholipid composition significantly alters K-Ras4B signaling [22].

Localization of K-Ras4B at the plasma membrane can be characterized by the quantity of its nanoclusters that act as signal transduction platforms. K-Ras4B requires phosphatidylserine (PtDSer) to form these nanoclusters [25]. K-Ras4B cannot interact with fully saturated PtDSer, and will not form nanoclusters without asymmetric PtDSer [25]. Mutational studies of the plasma membrane binding domain have shown that certain residues have large scale impacts on lipid composition of these nanoclusters. Specifically, mutations on K177/178Q lead to an increase in nanoclustering as a result of PIP2 binding rather than PtDSer [25].

The orientation of the HVR plays a role in binding between phospholipids and K-Ras4B [25,54]. Although there is no definitive structure of the different conformations that K-Ras4B can occupy, it has been concluded that the orientations change the availability of binding sites on the HVR. Since efficient function requires effective binding between phospholipids and the G-domain, varying orientations can block certain key residues from forming bonds and can drastically change the binding affinity and binding partners. Computational models have given rise to a few possibilities for what these orientation states may look like. Two major states are described: The first takes the form of a helical turn and extended backbone, while the second is an array of bent ensembles [24]. The majority of the population tends to fall into state 1. Other studies have found similar results, the existence of at least two states, although they have been defined by slightly different parameters. The mutational studies on the HVR showed that K177/178Q mutants most drastically changed the populations of orientation states [25].

Through interactions between the poly-basic domain and key phospholipids in the membrane, such as: PtDSer, PIP and PA, the HVR contacts the plasma membrane. The formation of nanoclusters is highly dependent on the integrity of the poly-basic domain and can severely impact signal transduction. The poly-basic domain of the HVR also impacts the orientation of the G-domain, which can significantly alter binding affinity for the plasma membrane and access to effectors. Simply put, the poly-basic domain of the HVR is the anchoring point of K-Ras4B to the plasma membrane and through a combination of clustering, orientation and phospholipid population, can efficiently regulate K-Ras4B function.

## 5. The HVR Undergoes Post-Translational Modifications

K-Ras4B function, membrane binding, and interactions with other proteins are regulated by post-translational modifications (PTMs) [56,57,58,59,60]. Several types of PTMs have been observed, including ubiquitination, acetylation, phosphorylation, carboxymethylation, and prenylation. Unlike other Ras proteins (H-Ras, N-Ras, and K-Ras4A), K-Ras4B is not palmitoylated, as it lacks the necessary cysteine in its HVR. PTMs of Ras proteins are found in both the G-domain (1–166 aa) and the HVR (167–188/189 aa). Ras proteins have similar G-domains but differ drastically in their HVR regions [60]. Therefore, it is reasonable to assume that HVR influences directly or indirectly the unique patterns of PTMs.

**Figure 2:**
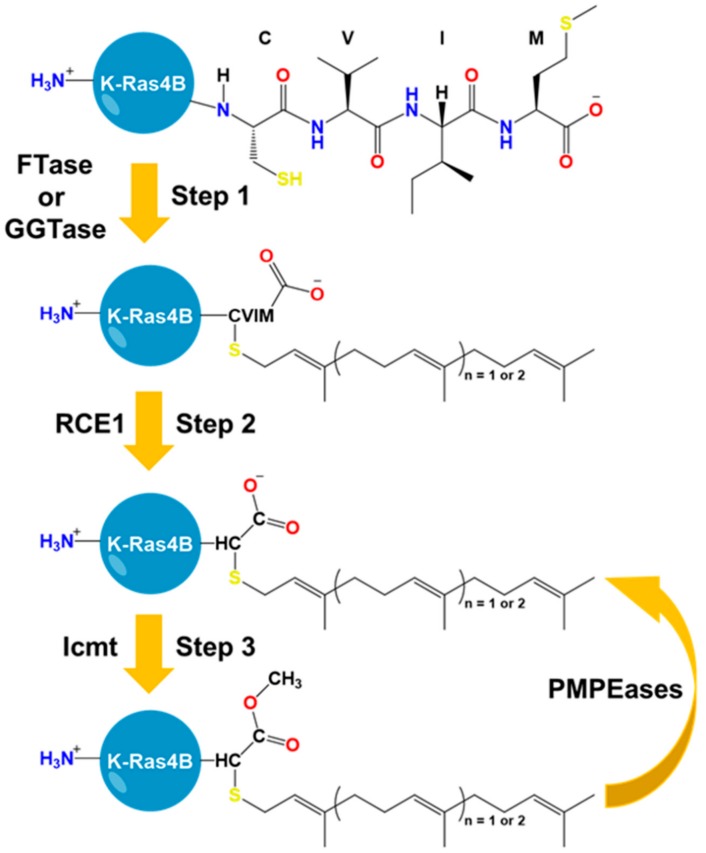
Prenylation, carboxymethylation and decarboxymethylation of K-Ras4B. The addition of a 15 carbon tail in the form of a farnesyl prenly group or 20 carbon tail in the form of a geranylgeranyl prenyl group to C185 is catalyzed by farnesyltransferase enzyme (FTase) and geranylgeranyltransferase (GGTase), respectively. This event is followed by two step reaction involving the hydrolysis of the–VIM portion of the CaaX motif by Ras converting enzyme 1 (RCE1) and the exposure of the carboxylic terminal of C185 for esterification by isoprenylcysteine carboxyl methyltransferase (Icmt). The latter step is a reversible step that can be catalyzed by carboxylesterases called Prenylated/polyisoprenylated methylated protein methyl esterases (PMPEases).

PTMs of the HVR of K-Ras4B are either irreversible or conditional [61,62]. The irreversible PTMs include farnesylation or alternative geranylgeranylation of the C-terminal C185 of the CVIM motif (Figure 2) [62,63]. The prenylation reaction proceeds through the addition of an isoprenyl group to the C185 side chain via a thioether bond. This step is catalyzed by cytosolic prenyltransferases (farnesyltrasferase (FTase) or geranylgeranyltransferase (GGTase)) (Figure 2). Farnesylated/geranylgeranylated K-Ras4B is transferred to the endoplasmic reticulum for further processing. The first event is cleavage of the last three amino acids, freeing the cysteine carboxyl terminus. This hydrolysis is catalyzed by the endopeptidase enzyme called Ras-converting enzyme 1 (RCE1) (Figure 2). The second event is the addition of a methyl group to the carboxyl terminus of C185, forming a reversible ester bond. This step is catalyzed by isoprenylcysteine carboxyl methyltransferase (Icmt) (Figure 2). The outcome of these modifications is to provide a hydrophobic density that can enhance the protein recruitment to the plasma membranes. Using top-down proteomic approaches, it was possible to quantify with high specificity the constitutive PTMs of K-Ras4B in cancer cells [64]. Depending on the tumor type and K-Ras4B mutation, there was a clear difference in the level of the reversible carboxymethylation of C185. In certain colorectal cell samples, oncogenic K-Ras4B can be found mostly free from carboxymethylation [64]. As the reaction is known to be reversible, it was of great interest to identify the possible enzymes involved. Prenylated/polyisoprenylated methylated protein methyl esterases (PMPEases) are a group of carboxylesterases that hydrolyze the ester terminal of prenylated substrates such as the members of the Ras family [65]. These enzymes were first identified in Dr. Lamango laboratory [66]. The PMPEases have highest affinity toward prenylated substrates compared to other esterases [65,67]. This implies the high selectivity of these enzymes toward prenylated substrates such as K-Ras4B and other Ras family members. Interestingly, PMPMEases was found to be hyperactive or/and overexpressed in Ras dependent cancer and their inhibitors can lead to their cellular death [68,69,70,71,72,73,74,75]. Hence, the interplay between Icmts and PMPMases can modulate the equilibrium of methylated/demethylated K-Ras4B population in tumors and consequently can impact downstream signaling, protein–protein interactions, or protein–lipid interactions.

The post-translational processing of the CaaX motif of K-Ras4B-HVR and its involvement in driving cancer has led to the development of inhibitors that target enzymes involved in PTM [61,62,76]. Discovery of FTase inhibitors (FTIs) was successful and many candidates were shown to be relatively nontoxic and to engage their target in vivo. However, in clinical trials, these inhibitors failed to inhibit mutant K-Ras4B-driven cancer. It was later found that GGTase can modify K-Ras and N-Ras but not H-Ras in the presence of potent FTIs, providing a hydrophobic density sufficient to bind the plasma membrane. Attempts to overcome this by targeting both prenyltranferases were unsuccessful. The dual acting inhibitors were very toxic and needed fine tuning of their therapeutic index that was found to be challenging. Another strategy is to target RCE1 and Icmt, in spite of the challenges regarding specificity and toxicity. Successful Icmt inhibitors were designed and had modest anti-tumor activity as a single therapy. Interestingly, these inhibitors can enhance the sensitivity to FTIs inhibitors and hence they can be used as combination therapy with FTIs. An alternative strategy was suicide inhibition by utilizing the current mechanism of prenylation by FTase [77]. This led to the design of electrophilic small molecule substrates that can modify the C185 and stop further processing of K-Ras4B. The new inhibitors had success in preventing K-Ras4B membrane localization in vitro, but they lacked selectivity to target oncogenic mutants of K-Ras4B vs wild type. Nevertheless, the strategy is a good start to discover small molecule inhibitors that can selectively modify the HVR of oncogenic K-Ras4B based on their dynamic structural differences.

One of the best known reversible PTMs in HVR is phosphorylation [59,61,76]. This PTM is mediated by at least two kinase enzymes: Protein kinase C (PKC) [16,58,78] and cyclic GMP-dependent protein kinase 2 (PKG2) [79]. Two serine residues at positions 171 and 181 in the HVR can be phosphorylated [58,59,78,80,81]. Phosphorylation of S171 is less vital for K-Ras4B function [78]. Therefore, most studies focused on phosphorylation of S181. Phosphorylated S181 operates a farnesyl-electrostatic switch that weakens K-Ras4B association with the plasma membrane, leading to redistribution to the cytoplasm and endomembranes [16,61,78,82]. Association of phosphorylated K-Ras4B (pK-Ras4B) with the plasma membrane can be dependent on the level of fluidity of the plasma membranes. In highly fluid plasma membrane domains, pK-Ras4B binds lipids tightly, whereas, weaker binding to highly dense lipid rafts occurs [82]. Thus, phosphorylation of K-Ras4B results in structural dynamic changes upon association or/and partitioning in the lipid domains based on lipid composition and level of fluidity [82,83]. In the context of protein–protein interactions, phosphorylation at S181 of K-Ras4B does not affect binding to its shuttle protein PDE-δ or its effector Raf-1 [80,82], while this event can regulate calmodulin binding to K-Ras4B [78,81]. Functionally, phosphorylation of K-Ras4B can have either a negative [16,84] or a positive [78,80] regulatory effect on the tumor cell growth, depending on the conditions. For instance, pK-Ras4B relocates to the mitochondria, endoplasmic reticulum, and Golgi body and, via association with the mitochondrial membrane protein Bcl-XL, leads to apoptosis [16]. On the other hand, pK-Ras4B can be essential for tumor cell growth and this is modulated by interaction with calmodulin [78,80,81]. In the dephosphorylated state, K-Ras4B binds calmodulin to prevent S181 phosphorylation, leading to short signaling [78,81]. Upon calmodulin inhibition, S181 is available for phosphorylation by PKC and this leads to prolonged signaling [78,81]. In summary, phosphorylation of the HVR of K-Ras4B can affect its function, membrane localization, and interactions with key regulatory proteins. However, the structural description of the effects of HVR phosphorylation are lacking.

Other conditional PTMs are possible. For instance, in a drug resistant model of chronic myeloid leukemia, cullin-based ubiquitin ligase 3 (CUL3)/leucine zipper-like transcription regulator 1 (LZTR1) complex inhibition leads to increased MAPK signaling and reduced sensitivity to chemotherapy [85]. These effects stem from the loss of ubiquitination of a conserved lysine residue in the Ras family (H-Ras, N-Ras, and K-Ras4A) at position 170 [86]. Ubiquitination of K170 negatively regulates oncogenic activity by dissociating Ras from the plasma membrane, reducing MAPK signaling, and enhancing sensitivity to chemotherapy [85,86]. Although K-Ras4B does not have a lysine at position 170, other lysines in the HVR might undergo ubiquitination.

The disordered HVR of K-Ras4B might be potentially affected by PTMs that have not yet been experimentally observed, such as acetylation, methylation, enzymatic and non-enzymatic glycosylation (glycation), and others [87,88,89,90]. The unique HVR region of K-Ras4B is characterized by the presence of eleven lysines. The poly-lysine region of K-Ras4B (175–188 aa) is vital for plasma membrane binding and cells transformation to malignancy [91]. This disordered area of lysines can be prone to acetylation [87,92], mono/di/tri-methylation [87,93], *N*-glycation [94] or/and mono/di-ubiquitination [87,95] as not all of them are involved directly in plasma membrane binding [25,87,91,96,97,98]. Additionally, the HVR region has residues that can be prone to *O*-glycosylation or/and phosphoglycation, such as S171, S181, and T183. Therefore, further investigation of currently known K-Ras4B-HVR-PTMs or/and identify novel PTMs for the unique HVR region can provide crucial structural and functional insights for development of specific therapies.

## 6. Conclusions

Because of its central role in cancer, K-Ras4B has been the focus of intense research for a long time. Tremendous progress has been made in understanding its structure and function and, importantly, the innovative approaches to therapeutically target K-Ras4B have been devised. These include covalent modification of the unique cysteine in oncogenic G12C K-Ras4B [99] and exploring novel binding pockets with low molecular weight compounds with the aim to inhibit GTP loading [100]. While reports on successful implementation of these strategies to help cancer patients are eagerly anticipated, the need to plan future K-Ras4B research is becoming more urgent. Undoubtedly, strategies to inhibit K-Ras4B in cancer will be optimized and diversified; to accomplish this, continued investigation of unique properties of K-Ras4B’s HVR will likely be essential.

The cationic nature and C-terminal modifications with farnesyl/geranylgeranyl and methyl groups in the HVR of K-Ras4B support its membrane binding function. At the plasma membrane, the HVR primarily interacts with negatively charged PtDSer [25] and phosphatidylinositols [55], while binding phosphatidic acid requires assistance of the G-domain and can be enhanced by oncogenic mutations [22]. The possibility that phosphatidic acid brings together K-Ras4B and its main GTP exchange factor son of sevenless provides a potentially important regulatory mechanism for aberrant activation of oncogenic K-Ras4B [101]. Other mechanisms employ regulation of effector binding by distinct orientations of the mutated G-domain on the plasma membrane and nanoclustering [23,102]. It seems possible to exploit unique protein–lipid and membrane regulated protein–protein interactions influenced by oncogenic mutations for drug design.

In addition to playing a major role in membrane targeting, the HVR of K-Ras4B can be sequestered by the G-domain to serve an auto-inhibitory function by reducing affinity for effectors [10]. The HVR sequestration may be regulated by nucleotide binding to prevent undesired or accidental signaling. Oncogenic mutations are predicted to impair this mechanism by altering the binding sites for HVR; however, more studies are needed to provide experimental evidence [21]. Development of small molecules or peptides capable of stapling the HVR back to the G-domain to abate effector binding and signaling might be an attractive approach to K-Ras4B inhibition.

**Figure 3:**
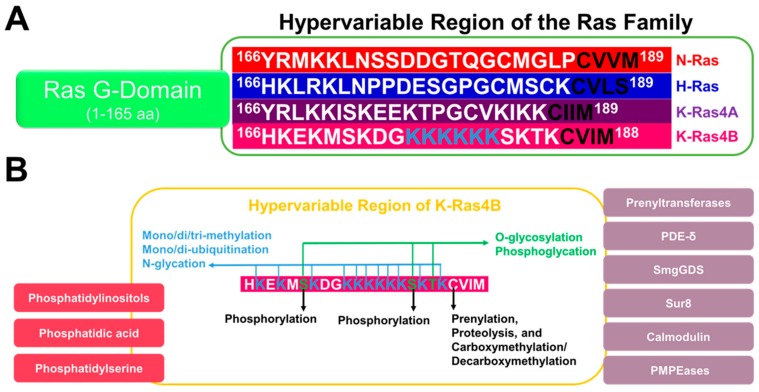
The hypervariable region (HVR) of K-Ras4B is unique and therefore it can influence several processes related to membrane binding and localization, protein–protein interactions, and posttranslational modifications (PTMs). (**A**) A cartoon of the Ras family proteins (N-Ras, H-Ras, K-Ras4A/B) shows the highly homologous G-Domain (1–165 aa) in green and the unique HVR of each isoform: N-Ras (Red); H-Ras (Blue); K-Ras4A (Purple); and K-Ras4B (Pink). The CaaX motif is shown in black. The unique polylysine region of K-Ras4B is colored light blue. (**B**) Key interactions of HVR with lipids (Rose), proteins (light Purple), and PTMs (Black) are highlighted. The predicted PTMs are shown in green and blue colors.

Accumulating evidence suggests that the HVR participates in Ras isoform-specific protein–protein interactions [103]. Because of its unique amino acid sequence (Figure 3), the HVR mediates binding of K-Ras4B to calmodulin, while H-Ras, N-Ras, and K-Ras4A do not bind [33]. Lysines in the HVR poly-basic region increase the affinity of K-Ras4B for farnesyltrasferase, ensuring its efficient prenylation [11], and together with the carboxymethyl group, enhance the interaction with tubulin [12], while binding to PDE-δ is primarily mediated by the farnesyl group [13]. HVR of K-Ras4B likely directs isoform-specific signaling through B-Raf [43], the scaffolding protein Sur8 [44], guanine nucleotide exchange factor and chaperone Smg-GDS [104], a lectin family member galectin 3 [47], vacuolar v-ATPase a2 and translation initiation factor eIF2Bδ [48], phosphatidylinositol-4-phosphate 5-kinase type 1α [49], and others, but this has not been investigated in sufficient detail and will be subject to future research efforts. We have similar expectations for PTMs in the HVR (Figure 3). So far, only classical prenylation [42], carboxymethylation [105], ubiquitination [86], and phosphorylation [15] have been described in depth, while multiple examples in the literature suggest that lysine-rich regions are frequent targets of acetylation [106], glycation [107], carbamoylation [108], and methylation [109]. However, these PTMs have not been observed in K-Ras4B. It will be important to find out if the HVR can also be modified through these processes or, if it cannot, why additional PTMs are prevented (Figure 3).

In summary, K-Ras is a very well-studied protein. However, there is still much to learn about its structure, dynamics, function, and ways of therapeutic targeting. We anticipate that significant advancement in the field will come from studies of the HVR, ultimately leading to the development of improved treatments for cancer patients.

## Abbreviations

K-Ras4B	Kirsten ras oncogene homolog B
HVR	Hypervariable region
PDE-δ	Phosphodiesterase δ
PKC	Protein kinase C
PKA	Protein kinase A
GDP	Guanosine diphosphate
GTP	Guanosine triphosphate
GEF	Guanine nucleotide exchange factor
GAP	GTPase-activating protein
PI3K	Phosphatidylinositol 3-kinases
aa	Amino acids
SWI	Switch I region
SWII	Switch II region
SmgGDS	A chaperone and a guanine nucleotide exchange factor
RASSF	Ras association domain-containing protein
IQGAP1	Q motif GTPase activating protein 1
PIP2	Phosphotidylinositol 4,5-Bisphosphate
PIP3	Phosphatidylinositol-trisphosphate
PA	Phosphatidic acid
PtDSer	Phosphatidylserine
PTMs	Post-translational modifications
FTAse	Farnesyltrasferase
GGTase	Geranylgeranyltransferase
GTPase	Guanosine triphosphate hydrolyzing proteins
RCE1	Ras-converting enzyme 1
Icmt	Isoprenylcysteine carboxyl methyltransferase
PMPEases	Prenylated/polyisoprenylated methylated protein methyl esterases
FTIs	FTase inhibitors
PKG2	Cyclic GMP-dependent protein kinase 2
GMP	Guanosine monophosphate
pK-Ras4B	Phosphorylated K-Ras4B
CUL3	Cullin-Based Ubiquitin Ligase 3
LZTR1	Leucine zipper-like transcription regulator 1
MAPK	Mitogen-activated protein kinase
